# Long-read sequencing improves the genetic diagnosis of retinitis pigmentosa by identifying an Alu retrotransposon insertion in the *EYS* gene

**DOI:** 10.1186/s13100-024-00320-1

**Published:** 2024-05-04

**Authors:** Elena Fernández-Suárez, María González-del Pozo, Cristina Méndez-Vidal, Marta Martín-Sánchez, Marcela Mena, Belén de la Morena-Barrio, Javier Corral, Salud Borrego, Guillermo Antiñolo

**Affiliations:** 1 Department of Maternofetal Medicine, Genetics and Reproduction, Institute of Biomedicine of Seville (IBiS), University Hospital Virgen del Rocío/CSIC, University of Seville, Seville, Spain; 2 Center for Biomedical Network Research On Rare Diseases (CIBERER), Seville, Spain; 3 Servicio de Hematología y Oncología Médica, Hospital Universitario Morales Meseguer, Centro Regional de Hemodonación, Universidad de Murcia, IMIB-Pascual Parrilla, CIBERER-ISCIII, Murcia, Spain

**Keywords:** Alu insertion, CNV, *EYS*, Inherited retinal diseases, Long-read sequencing, Nanopore sequencing, Retinitis pigmentosa, Retrotransposon

## Abstract

**Background:**

Biallelic variants in *EYS* are the major cause of autosomal recessive retinitis pigmentosa (arRP) in certain populations, a clinically and genetically heterogeneous disease that may lead to legal blindness. *EYS* is one of the largest genes (~ 2 Mb) expressed in the retina, in which structural variants (SVs) represent a common cause of disease. However, their identification using short-read sequencing (SRS) is not always feasible. Here, we conducted targeted long-read sequencing (T-LRS) using adaptive sampling of *EYS* on the MinION sequencing platform (Oxford Nanopore Technologies) to definitively diagnose an arRP family, whose affected individuals (*n* = 3) carried the heterozygous pathogenic deletion of exons 32–33 in the *EYS* gene. As this was a recurrent variant identified in three additional families in our cohort, we also aimed to characterize the known deletion at the nucleotide level to assess a possible founder effect.

**Results:**

T-LRS in family A unveiled a heterozygous AluYa5 insertion in the coding exon 43 of *EYS* (chr6(GRCh37):g.64430524_64430525ins352), which segregated with the disease in compound heterozygosity with the previously identified deletion. Visual inspection of previous SRS alignments using IGV revealed several reads containing soft-clipped bases, accompanied by a slight drop in coverage at the Alu insertion site. This prompted us to develop a simplified program using *grep* command to investigate the recurrence of this variant in our cohort from SRS data. Moreover, LRS also allowed the characterization of the CNV as a ~ 56.4kb deletion spanning exons 32–33 of *EYS* (chr6(GRCh37):g.64764235_64820592del). The results of further characterization by Sanger sequencing and linkage analysis in the four families were consistent with a founder variant.

**Conclusions:**

To our knowledge, this is the first report of a mobile element insertion into the coding sequence of *EYS,* as a likely cause of arRP in a family. Our study highlights the value of LRS technology in characterizing and identifying hidden pathogenic SVs, such as retrotransposon insertions, whose contribution to the etiopathogenesis of rare diseases may be underestimated.

## Background

Inherited retinal dystrophies (IRD) are a group of clinically and genetically heterogeneous pathologies characterized by photoreceptors or retinal pigment epithelial cell dysfunction leading to irreversible and progressive visual impairment [1]. To date, variants in more than 300 genes and *loci* have been associated with autosomal-recessive, autosomal-dominant, X-linked, and mitochondrial inheritance (https://web.sph.uth.edu/RetNet/ accessed on October 2023), showing the wide heterogeneity of these disorders [2]. The most common form of IRD is retinitis pigmentosa (RP, ORPHA:791), which affects more than 1.5 million patients worldwide (1:4000) [3]. RP typically manifests with night blindness as the first symptom, reflecting the principal dysfunction of rod photoreceptors, followed by concentric visual field loss, and a decrease in visual acuity due to secondary cone dysfunction [3]. Some of the most commonly mutated genes in non-syndromic RP include *RHO* [4]*, USH2A* [5], *EYS* [6–8], or *RPGR* [9].

Currently, short-read sequencing (SRS) is the most commonly used approach to genetically diagnose RP patients in clinical routine, enabling the identification of disease-causing variants in an estimated 60% of cases [10, 11]. Although the application of recent advances, such as whole-genome sequencing (WGS), has the potential to increase the diagnostic yield of IRD patients by facilitating the identification of copy number variations (CNVs), and allowing the detection of variants in GC-rich regions, non-coding regions [12–14], or in novel candidate disease genes [15, 16], a substantial number of patients remain without genetic diagnosis [13, 14]. This may be due to the shortcomings of SRS in detecting complex structural variants (SVs) [17], such as, mobile element insertions (MEIs), inversions, or translocations, which have previously been associated with the etiopathogenesis of the IRD [18–20].

In this sense, the arrival of long-read sequencing (LRS) technologies has raised great expectations about their potential to discover unknown etiological variants. Despite the relatively high sequencing error rate, LRS allows more accurate detection and characterization of SVs, overcoming some of the limitations of SRS [21]. Long-read genome sequencing also offers clear advantages in the detection of the physical phasing of genomes and methylation differences, which are simultaneously detected without needing additional experiments [22]. However, this approach still has certain disadvantages that hamper its use by human genetics researchers and clinicians, including high costs, low throughput, computational overhead, and the lack of large databases for LRS data interpretation [22, 23]. To reduce costs and simplify the analysis, targeted LRS (T-LRS) has been shown to be effective in identifying missing variants in specific genes of interest [24]. Nanopore sequencing, thanks to its flexibility of data acquisition with real-time analysis, allows target enrichment by directly rejecting or accepting DNA molecules during sequencing without specific sample preparation [25]. This in silico enrichment, termed adaptive sampling, avoids the sequencing of uninformative or off-target reads, thereby increasing the depth of coverage of the target region [24, 26]. While targeted LRS is useful for the study of genetic diseases caused by mutations in a single gene [27, 28], for more heterogeneous diseases, like IRD, the selection of partially solved patients carrying a monoallelic likely causative variant in an autosomal recessive gene is key to increase the success rate by focusing the analysis on a single genomic region. In this sense, a good candidate to be explored by LRS would be the *EYS* gene*,* in which has been described that CNVs are a relatively common type of genomic rearrangement [29–31]. *EYS* is one of the largest genes expressed in the retina, spanning over 2Mb of genomic DNA [6, 7, 32],and is one of the most prevalent genes in autosomal recessive RP (arRP) in diverse populations [7, 8, 33]. Remarkably, in the HGMD-pro database (accessed on October 2023), a large number of pathogenic/likely pathogenic variants (*n* = 749) are reported, of which 87 correspond to gross deletions and insertions.

Here, the application of T-LRS using adaptive sampling in a patient with arRP carrying a pathogenic deletion of exons 32–33 in the *EYS* gene allowed us to fulfill the complete molecular diagnosis 4 years after the firsts analysis, because we identified an insertion of 352bp Alu repeat sequence in the coding sequence of exon 43 as a potential second causative variant of arRP in this family. Moreover, T-LRS enabled us to determine that both defects were in different alleles and to define the breakpoints of the aforementioned *EYS* deletion. To our knowledge, this is the first time that a mobile element insertion in the *EYS* gene has been reported as a disease-causing variant, enlarging the number of genes affected by this pathogenic mechanism.

## Materials and methods

### Subjects, clinical evaluation, and previous studies

One Spanish family consisting of 8 unaffected and 3 affected individuals with a presumed arRP, was recruited for genetic diagnosis (Family A). Moreover, two additional genetically solved arRP families (Families B and C) and one unclassified IRD family carrying the heterozygous deletion of exons 32–33 of *EYS* were included in this study. Peripheral blood was collected from the subjects to extract genomic DNA (gDNA) using standard procedures. An informed consent form was signed by all participants or their legal guardians for clinical and genetic studies. Experiments were conducted according to the principles of the Declaration of Helsinki (Edinburgh, 2000) [34], and approved by the Institutional Review Boards of the University Hospital Virgen del Rocio and the University Hospital Virgen Macarena (Seville, Spain).

As part of our diagnostic routine, the proband of family A underwent targeted sequencing of a custom panel that included all coding exons and the splice junctions of 1,166 genes associated with different rare diseases [35, 36]. The sequencing was performed on the NextSeq500 instrument (Illumina, San Diego, CA, USA). Single nucleotide variants (SNVs) and indels were analyzed using a corporate prioritization tool, whereas an in-house independent script based on coverage and statistical studies was used for the analysis of CNVs [35]. Briefly, the prioritization of SNVs and indels was done as following: i) application of a virtual panel of 146 IRD genes; ii) frequency filtering, *minor allele frequency* (MAF) below 0.01 in 1000GP, the Exome Aggregation Consortium (ExAC), the Genome Aggregation Database (GnomAD), Exome Variant Server (EVS); and iii) consequence filtering: coding nonsynonymous variants and splice variants (8 bp intronic and 2bp exonic). As for CNVs, the prioritization of the variant was done according to the highest absolute values of the z-score. In addition, we used the Mobile Element Locator Tool (MELT v2.2.2) [37] to discover mobile element insertions (Alu, L1, and SVA elements).

The CNV affecting *EYS* were previously analyzed and validated by Multiplex Dependent Probe Amplification (MLPA) with SALSA MLPA Probe mix P328-A3 (MRC Holland). The MLPA reactions were run on ABI 3730 DNA Analyzer (Applied Biosystems) and the data was evaluated using GeneMarker v.1.75 (SoftGenetics) as previously described [30].

### Long‑read sequencing and data processing

For nanopore sequencing, we used the sequencing service provided by LongSeq Applications (Murcia, Spain) using the MinION device (Oxford Nanopore Technologies). Briefly, approximately 2,305 ng of gDNA was used to prepare the sequencing library using the ONT Ligation Sequencing Kit (SQK-LSK109) following the manufacturer’s protocol with slight modifications. Bead-based washes were performed using Low Fragment Buffer and the final library was eluted in 15 µl of Elution Buffer, following a 10 min incubation at 37ºC. Approximately, 200 ng of DNA library were loaded onto a MinION Flow Cell (R9.4.1). *EYS* enrichment was performed using adaptive sampling tool, implemented in the MinKNOW software (ONT) [38], whose input was the FASTA file obtained from this genomic coordinate: chr6:63783736–66808386 (GRCh37/hg19). Sequencing experiment were run for up to ~ 40h.

Bioinformatic analysis of LRS data was performed as previously described [27] and consisted of: i) base calling using Guppy, which is integrated within the MinKNOW software [38]; ii) alignment to the human reference assembly (GRCh37/hg19) using Minimap2 [39, 40]; iii) variants calling with Sniffles software for SVs [41] and Clair3 for SNVs [42]. The SVs file was annotated using AnnotSV v3.3.6 [43], whereas the VCF file containing SNVs was annotated with Alamut Batch v1.11 and SnpEff v5.1 [44] to add the SpliceAI and CADD v.1.6 scores. The prioritization of SNVs detected by ONT was conducted using our pipeline as previously described [15, 36]. For SVs analysis, variants involving coding exons were prioritized.

### Validations and breakpoints sequence analysis

In the index patients from the four families, the breakpoints of the *EYS* exons 32–33 deletion were assessed by PCR using mutation-specific primers: 5’-CCTTTACAAGACATGAGCATGCTGGGA-3’ (intron 33, forward) and 5’-ATTCCTTACTCCCTAGCCCTGCTGTAA-3’ (intron 31, reverse). The amplification reaction was performed using Multiplex PCR Master Mix (Qiagen) followed by 35 cycles of 94 °C for 30 s, 60 °C for 90 s, and 72 °C for 90 s. Under these conditions, only the mutant allele can be amplified, as the wild-type allele is larger (~ 56kb).

The validation of the *EYS*-Alu insertion in family A was performed by PCR using mutation-specific primers designed using the information obtained by nanopore sequencing: 5’-TTTTAGCCGGGATGGTCTCGATCTCC-3’ (AluYa5, forward) and 5’-GAGAAACCTCCAGTTCACTACTATATCC-3’ (exon 43, reverse) for the 5’-junction and 5’-TGTAGGAAAAACAATCAGAACCTTCAGTG-3’ (exon 43, forward) and 5’-GGAGATCGAGACCATCCCGGCTAAAA-3’ (AluYa5, reverse) for the 3’-junction. The PCR reaction was performed using NXT Taq PCR Master Mix 2X (EURx Ltd.) according to the manufacturer’s protocol. The amplification conditions were the following: 95°C for 5 min; 35 cycles of 95°C for 5 s, 60°C for 5 s and 68°C for 40 s; final extension at 68°C for 30 s.

PCR products were analyzed using the QIAxcel capillary electrophoresis system and QIAxcel ScreenGel software (Qiagen) [45]. Sanger sequencing was performed after PCR cleanup (ExoSap-IT, Affymetrix, Santa Clara,CA, USA) and sequenced (BigDye® Terminator v3.1 Cycle Sequencing Kit, 3730 DNA Analyzer, Applied Biosystems, USA)) using the primers described above.

### Linkage analysis by short tandem repeat markers

To assess a putative founder effect of the CNV-deletion of exons 32–33 of *EYS*, linkage analysis using short tandem repeat (STR) markers was carried out in 19 individuals from the four unrelated families, 14 of which carried the CNV. For this purpose, a total of eight STR markers flanking *EYS* were selected from the literature [32] and UCSC Genome Browser (D6S1573, D6S402, D6S1658, D6S1026, D6S1670, D6S430, D6S1557 and D6S1681). Multiplex PCR Master Mix (Qiagen) was employed to amplify and label the interest regions. PCR products were genotyped using 3730 DNA Analyzer (Applied Biosystems). The results were analyzed by GeneMapper v.4.0 software (Applied Biosystems).

### Screening for EYS-Alu insertion in SRS data

In order to evaluate the recurrence of the inserted Alu in our population, the Linux grep command (Table 1) was used to search in compressed FASTQ for the junctions between *EYS* exon 43 reference sequences and the beginning/end of the Alu insertions in the previously generated short-read NGS data of 327 additional individuals, including 149 unaffected and 178 IRD patients, as described elsewhere [46, 47]. For this purpose, we used four chimeric sequences of 23 nucleotides in length, containing the two boundaries of the insert (*EYS*-Alu and Alu-*EYS*) at both forward and reverse (Table 1). These sequences were observed by both LRS and Sanger in the index of family A, and were found to be specific for the mutant allele, as they did not match any other region of the genome. The wild-type sequence was also searched to determine the genotype of the Alu insertion. The grep commands returned the number of reads containing the matching sequences in each file, whose value was dependent on the coverage depth in that area. After the screening, the variant allele frequency (VAF) was calculated as mutant alleles/total alleles.

**Table 1:** Command line to search for the AluYa5 insertion in coding exon 43 of the EYS gene using the grep search program

Allele	FASTQ files	BAM files
WT	zgrep -c “ATTAGAAGGATACAATGT TTATG\|CATAAACATTGTATCCTTCTAAT” *fastq.gz > output.txt	samtools view sample1.bam | grep -c -e ATTAGAAGGATACAATGTTTATG -e CATAAACATTGTATCCTTCTAAT > output.txt
MUT	zgrep c- “GAAGGATACAATGTTGGCCGGGC\ |GCCCGGCCAACATTGTATCCTTC\| ACATTGTATCCTTCTTTTTTTT\| AAAAAAAAGAAGGATACAATGT” *fastq.gz > output.txt	samtools view sample1.bam | grep -c -e GAAGGATACAATGTTGGCCGGGC -e GCCCGGCCAACATTGTATCCTTC -e ACATTGTATCCTTCTTTTTTTT -e AAAAAAAAGAAGGATACAATGT > output.txt

## Results

### LRS data quality

Nanopore sequencing of the proband of family A using a MinION device yielded 8.25Gb with 9,832,520 total reads. After quality analysis by *qualimap*, 936,188 reads remained with a mean read length of 2,876kb and a maximum read length of ~ 110kb. The percentage of mapped reads across the reference genome was 98.66% with a mean mapping quality of 59.68. The mean coverage in the region of interest (chr6:63,783,736–66,808,386) was 7.0x ± 2.7 (mean ± SD).

### Identification of a novel mobile element insertion

**Fig. 1:**
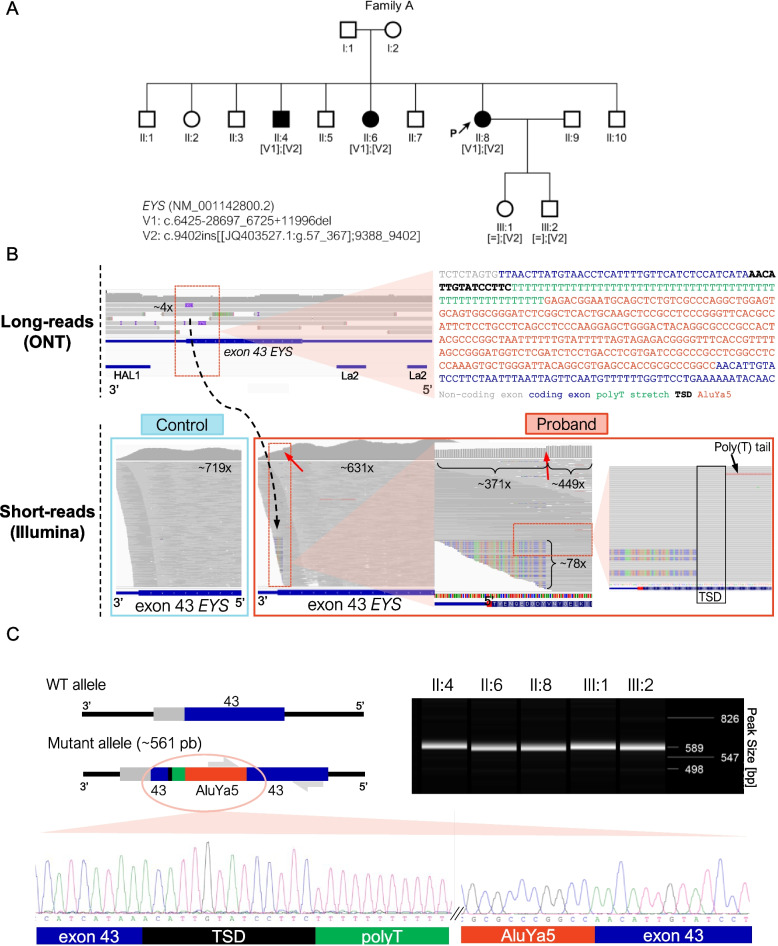
Identification and characterization of the AluYa5 insertion in the *EYS* gene. **A** Segregation analysis of identified variants in family A. Index patients are indicated with a black arrow and the letter P. **B** IGV screenshot of long-read sequencing data showing the sequence of the inserted element (282 bp Alu, 56 bp poly-T and 15 bp duplication of exon 43 sequence). Visual inspection of the short-read NGS data in IGV with the option “show soft-clipped bases” at the *Alu* insertion site of the proband from family A revealed multiple reads with aberrant alignments corresponding to the AluYa5 insertion. The space between the Alu insertion and the poly(T) tail corresponds to the target site duplication (TSD, sequence: AACATTGTATCCTTC). A slight drop in coverage at the junction of the Alu insertion is observed in the short-read NGS data (red arrow). **C** Validation of the junction of the AluYa5 insertion by PCR and Sanger sequencing

Index patient from family A received a clinical diagnosis of arRP (Table 2). In previous studies, targeted SRS in the proband of family A only allowed the identification of a heterozygous deletion of exons 32–33 in the *EYS* gene, which was also detected in both affected siblings (Fig. 1). Therefore, it was necessary to identify a second disease-causing variant to complete the genetic diagnosis in this family.

**Table 2:** Clinical characteristics of the index patients of the families carrying the *EYS* exons 32–33 deletion

Pedigree subject	Clinical diagnosis	Onset age	First symptom	Symptoms at time of the genetic assessment	Fundus examination
Fam. A -II:8 (female)	RP	15 years	Night blindness and reduction of the visual field	ERG consistent with RP; decreased of visual acuity	Narrowed vessels; bone spicule pigmentation
Fam B – II:1 (male)	RP	17 years	Night blindness and reduction of the visual field	Concentric reduction of the visual field (10º central);	Bone spicule pigmentation; Pallor of the optic disc Narrowed vessels; Foveal preservation; ERG consistent with RP
Fam C - III: 4 (female)	RP	35 years	Concentric reduction of the visual field	Night blindness; Concentric reduction of the visual field (10º central); Decreased visual acuity; Cataracts	Narrowed vessels; Pallor of the optic disc; ERG: No responses
Fam D - II:1 (female)	Unclassified IRD	17 years	Night blindness	Photophobia. Decreased visual acuity	No alterations

*Abbreviations*: *ERG* Electroretinography, *Fam* Family, *IRD* Inherited retinal dystrophy, *RP* Retinitis pigmentosa

Nanopore sequencing revealed 12 additional SVs and 325 rare (MAF < 0.01) SNVs/indels in *EYS.* Among the SVs, a heterozygous insertion of ~ 352bp in coding exon 43 was first prioritized in this family because it was the only one with an exonic breakpoint, and it was in a different phase than the previously detected deletion (Fig. 1). LRS provided the complete nucleotide sequence of the insert, which was analyzed using BLAST. The insert sequence included an AluYa5 element belonging to the SINE1/7SL non-LTR retrotransposon class [48], a poly(T) tail of 56 bp, and the characteristic target site duplication (TSD, sequence: AACATTGTATCCTTC) (Fig. 1). The Alu insertion disrupting the coding exon 43 would, if translated, result in an insertion of 39 aberrant amino acids followed by a premature termination codon.

The Alu repeat mobile element insertion was validated by mutant allele-specific PCR and Sanger sequencing (Fig. 1). Family segregation studies in additional family members confirmed that the *Alu* insertion segregated with the disease in the family in combination with the deletion of exons 32–33 and was transmited to the third generation (Fig. 1).

In previous targeted SRS studies of this family, the Alu insertion could not be identified, even after applying the MELT software. However, subsequent visual IGV inspection of this data revealed a number of reads with soft-clipped bases in the breakpoint region (76 reads out of 449 total reads; ~ 17%) (Fig. 1). The analysis of the bases was coincident with the AluYa5 sequence. In addition, a drop in coverage at the insertion point was observed, indicating that the standard BWA-based alignment of Illumina reads may fail to map chimeric reads (Alu*-EYS)* (Fig. 1).

### Screening for the EYS-Alu insertion in additional patients and controls using SRS data

Since the exon 43 of *EYS* was already included in our diagnostic panel, a command line based on grep was first validated using the SRS data of the index patient of family A (Table 1). In order to determine the recurrence and the prevalence of this Alu insertion in our cohort, the optimized command was applied for the screening of FASTQ files from 327 individuals, including 178 IRD patients and 149 unaffected individuals. However, none harbored the Alu insertion, supporting its low frequency, and thus, its pathogenicity. Taken together, these data led us to consider the AluYa5 insertion in exon 43 of *EYS* (chr6(GRCh37):g.64430524_64430525ins352, NM_001142800.2:c.9402_9403ins[[JQ403527.1:g.57_367];9388_9402]) as the most likely disease-causing second hit in the family A.

### Characterization of structural variants by LRS

**Fig. 2:**
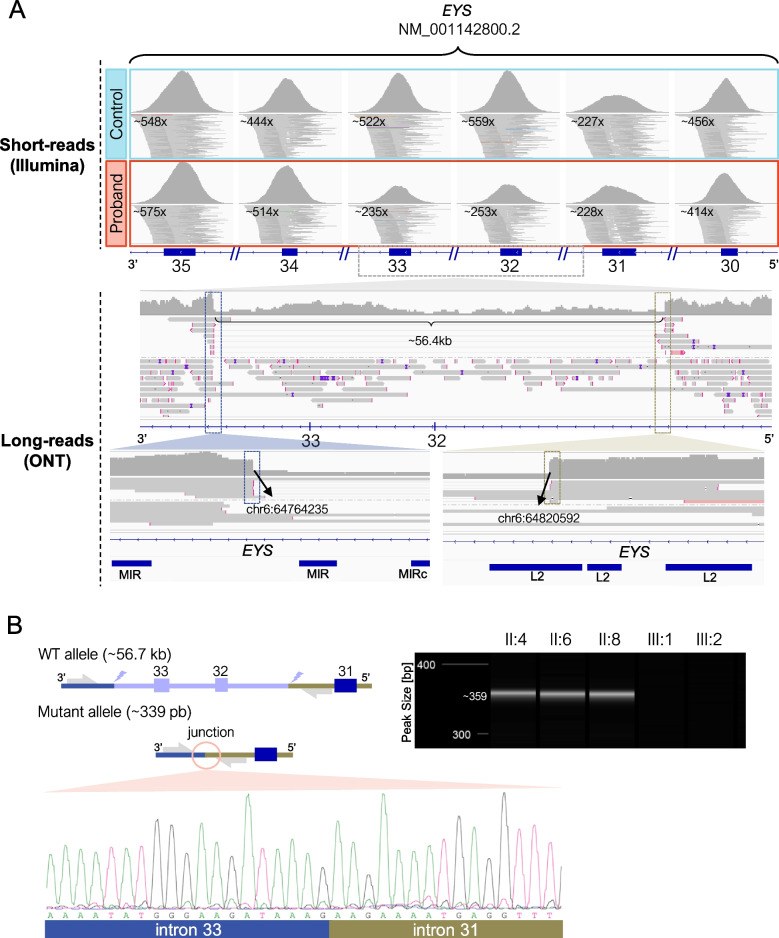
Characterization of the deletion of exons 32–33 in the *EYS* gene. **A** Integrative Genomics Viewer (IGV) screenshots showing the deletion of exons 32–33 in the *EYS* gene detected by short-read sequencing and its characterization by long-read sequencing. **B** Validation by PCR and Sanger sequencing of the deletion junction in all individuals of family A

In addition, nanopore sequencing using adaptive sampling in the index patient of family A enabled the characterization of the previously identified *EYS* CNV at nucleotide-level resolution with a 10 × coverage (Fig. 2), defining a ~ 56.4kb deletion that included exons 32–33 of *EYS* (chr6(GRCh37):g.64764235_64820592 del, NM_001142800.2:c.6425-28697_6725 + 11996del). Visual inspection in IGV software using the RepeatMasker database, allowed us to locate the 5’ breakpoint of the CNV in a long interspersed element (LINE) belonging to the L2 family of intron 31, whereas the 3’ breakpoint was flanked (~ 200bp upstream and 600bp downstream) by a short interspersed element (SINE) belonging to the mammalian interspersed repeats (MIR) family (Fig. 2). To verify the breakpoint junctions, mutation-specific primers were designed using the information obtained by LRS in the flanking regions of the CNV, allowing amplification only in the three affected siblings carrying the deletion (Fig. 2). Sanger sequencing confirmed the deletion breakpoints previously detected by nanopore sequencing.

**Fig. 3:**
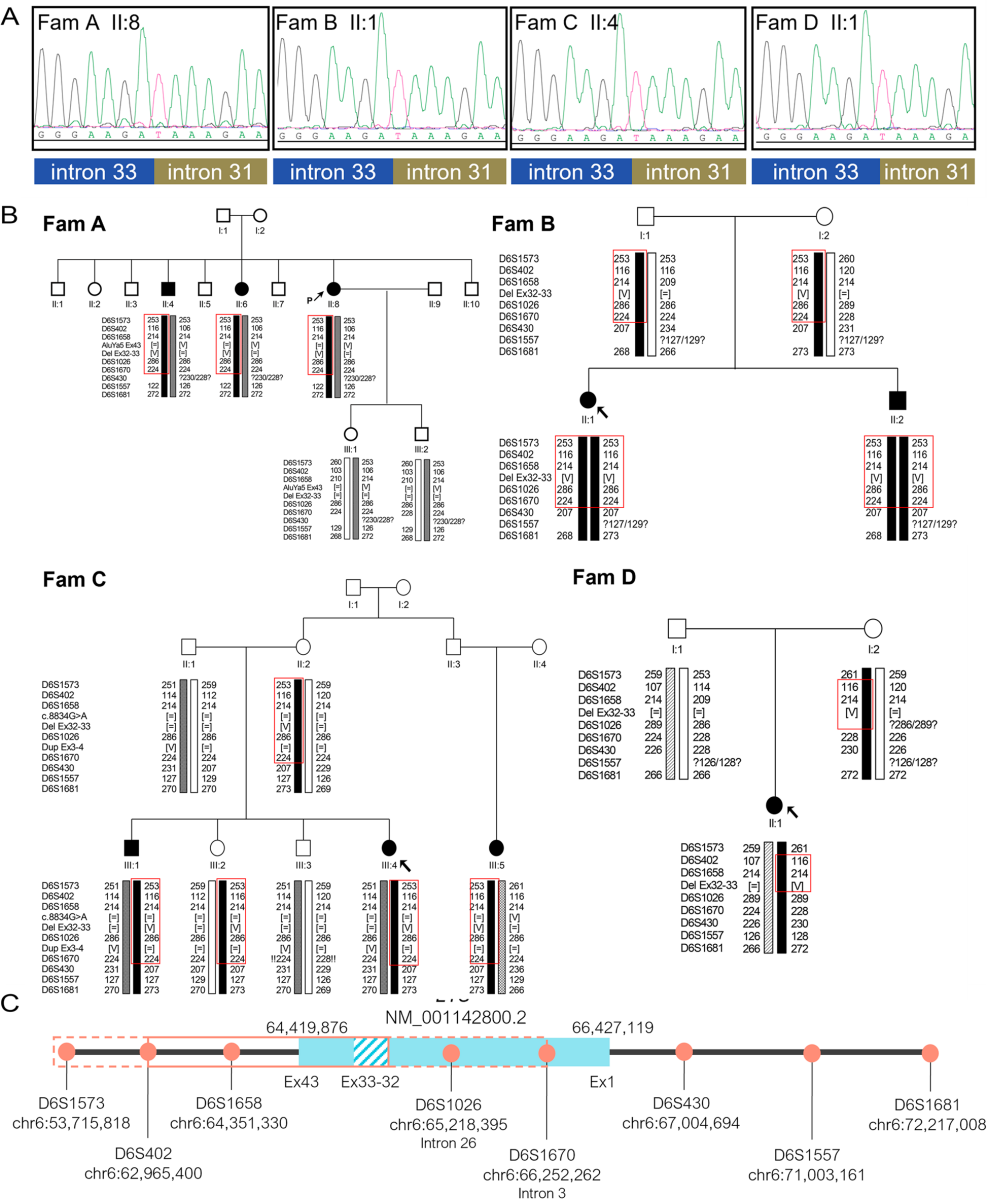
Linkage analysis of the four IRD families carrying the deletion of exons 32–33 of *EYS*. **A** Sanger sequencing of the deletion junction in the four index patients. **B** STR markers analysis in the 19 individuals from the four families. The allele carrying the deletion is shown in black. The region linked in each family is represented by a red square. **C** Representation of the selected STR markers, their chromosomal positions (GRCh37) and the putatively linked interval

Since a similar deletion involving exons 32–33 of *EYS* had previously been detected in three additional IRD families from our cohort (Table 2)*,* we performed Sanger sequencing and confirmed that they share the same breakpoints (Fig. 3). Then, microsatellite analysis revealed that the four families shared a region of ~ 1,9Mb, which extended up to ~ 12,5 Mb in families A, B and D (Fig. 3).

## Discussion

Sequencing technologies have evolved rapidly since the discovery of Sanger sequencing over 50 years ago. However, despite these advances, the diagnostic yield for IRD remains in the range of 52 to 74%, depending on the phenotype [10, 49], indicating that a substantial proportion of causative variants remain unidentified or misinterpreted. Therefore, a closer look at the detection of complex or non-coding variants that currently elude diagnostic pipelines would be of great benefit in increasing the diagnostic yield in IRD patients.

Here, we used LRS to identify the insertion of an AluYa5 element in the *EYS* coding exon 43 as one of the disease-causing variants in one arRP family, which was missed by a previous SRS approach. Alu elements are ∼300 bp sequences belonging to a class of mobile elements or retrotransposons called SINEs that comprise 11% of the human genome, with nearly a million copies located primarily in introns and intergenic regions [50]. Among the Alu subfamilies, AluYa5 and AluYb8 are currently the most active subfamilies in the human lineage [51]. Although retrotransposons have contributed in many ways to genetic and functional diversity during evolution, their insertion can also be deleterious, disrupting coding exons or key regulatory elements, and serving as substrates for non-allelic recombination leading to CNVs [52]. In fact, the role of MEIs in the etiopathogenesis of a significant number of Mendelian diseases, including IRD, have already been described [19, 46, 53]. Examples of this are the recurrent retrotransposon insertions in *MAK* [19], *RP1* [54] or *BBS1* [55], which were first serendipitously discovered in linked families from populations with strong founder effects. Interestingly, the poly(T) tail of the identified AluYa5 in *EYS* is longer than 50 bp, indicating that the insert is quite young. In fact, the A-tails of very recent Alu insertions have been described to be between 40 and 97 bp in length [56]. Long A-tails tend to shorten relatively quickly towards 30 bases in terms of generations [56], which could be consistent with the variant being private to the family in study. The identified variant is inserted within the coding sequence of the *EY*S gene [6, 32], which is often the target of SVs, mainly CNVs [30, 31]. Examination of the recently updated gnomAD SVs v4 database revealed the presence of two Alu insertions with different breakpoints disrupting the same coding exon in two heterozygous carriers (INS_CHR6_ 9AB69B96 and INS_CHR6_DD0F655F). The identification of three independent Alu insertions within the same exon may indicate a site of high susceptibility to these events. This would make the *EYS* gene a good candidate to explore for the identification of Alu insertions as disease-causing variants.

In addition, thanks to the capability of LRS to cover entire SVs, we characterized at the nucleotide level a recurrent CNV-deletion comprising exons 32–33 of *EYS*. This variant has been reported in the literature in Portuguese, Spanish and French arRP families [29, 30, 33, 57], as well as in three heterozygous carriers (MAF = 2.38e-05) from the recently updated gnomAD SVs v4 database from diverse genetic ancestry groups (Middle Eastern, Admixed American and African) (Variant ID: DEL_CHR6_4F5408B3). Moreover, we identified the same deletion in three unrelated Spanish families from our IRD cohort. In this study, the linkage analysis results were consistent with a founder effect variant that may have originated in the Iberian Peninsula and spread to other regions, although studies in other populations are needed. The importance of identifying prevalent founder variants is increasing, as they are potential candidates for variant-specific therapies that may benefit a larger number of patients. These therapies may include antisense oligonucleotides (AONs), which allow targeting of large genes that, as *EYS*, cannot be treated with conventional gene supplementation therapies [58]. Regarding large deletions, AONs can modulate pre-mRNA splicing, and restore the disrupted reading frame. This is the case of Eteplirsen, the drug approved for Duchenne muscular dystrophy [59, 60].

Our results are in line with previous studies [61, 62] that suggest that the impact of complex SVs may be underrepresented in a variety of Mendelian disorders, reinforcing the need of applying systematic detection methods of MEIs, especially in partially or completely unsolved cases. While some SVs, such as deletions or duplications of one or more coding exons, are easy to identify computationally using targeted SRS data, other SVs, such as MEIs, are much more technically challenging due to their genome-wide distribution and related alignment difficulties in SRS data on repetitive regions, requiring the use of specific calling algorithms preferably applicable to WGS [63]. In fact, in our hands, MELT failed to identify the insertion of the Alu element using previous targeted SRS data. However, most clinical genetics centers still prefer to use targeted SRS in their diagnostic routine due to its robustness and cost-effectiveness, which means that the full landscape of SVs may not be fully undercovered. Therefore, there is an urgent need to develop feasible strategies to detect these SVs in the clinical setting without investing in additional costly WGS protocols [61]. In this work, we proposed a re-analysis strategy to uncover specific *EYS*-AluYa5 insertion on available SRS data (FASTQ and/or BAM files) using the Linux grep command as previously described [46, 47].

As we have shown, multiple types of SVs, including a MEI and a CNV, can be detected simultaneously by enriching a particular genomic region by LRS using the ONT adaptive sampling method. This method is powerful and can be performed in a cost-efficient manner using a Nanopore MinION flowcell, which ensures sufficient depth of coverage while reducing problems associated with the introduction of PCR artifacts and PCR length restrictions [64]. It also allows to gather additional clinically relevant information such as the precise SVs breakpoints, the full sequence of the inserted Alu, and the phasing of the two compound heterozygous variants, which together may result in the resolution of heterogeneous genetic traits.

## Conclusions

In conclusion, our research has explored the potential of LRS to increase the diagnostic yield of an unsolved arRP family. As a result, adaptive sampling of the *EYS* gene revealed the precise location of a previously known recurrent CNV, as well as a novel Alu insertion both of which segregated with the disease in the family. In addition, haplotype analysis of unrelated families harboring the recurrent CNV was consistent with a founder variant, which may open the door to genomic medicine approaches in these patients. This is the first report of a pathogenic Alu insertion in the *EYS* gene, expanding its genotypic spectrum and strengthening the role of MEIs in the etiopathogenesis of IRDs.

## Data Availability

The datasets for this article are not publicly available due to concerns regarding participant/patient anonymity. Requests to access the datasets should be directed to the corresponding authors. The novel MEI was submitted to ClinVar database under the accession ID: SCV004697438.

## Abbreviations

AONs: Antisense oligonucleotides
arRP: Autosomal recessive retinitis pigmentosa
CNV: Copy number variations
EVS: Exome Variant Server
gDNA: Genomic DNA
GnomAD: Genome Aggregation Database
IGV: Integrative Genomics Viewer
IRD: Inherited retinal dystrophies
LINE: Long interspersed element
LRS: Long-read sequencing
MAF: Minor allele frequency
MEIs: Mobile element insertions
MELT: Mobile Element Locator Tool
MIR: Mammalian-wide interspersed repeats
MLPA: Multiplex Dependent Probe Amplification
ONT: Oxford Nanopore Technologies
RP: Retinitis pigmentosa
SINE: Short interspersed element
SNVs: Single nucleotide variants
SRS: Short-read sequencing
SVs: Structural variants
TSD: Target site duplication
VAF: Variant allele frequency
WGS: Whole-genome sequencing
